# The influence of a hot environment on physiological stress responses in exercise until exhaustion

**DOI:** 10.1371/journal.pone.0209510

**Published:** 2019-02-06

**Authors:** Romeu P. M. Silva, Cristiano L. M. Barros, Thiago T. Mendes, Emerson S. Garcia, Vitor E. Valenti, Luiz Carlos de Abreu, David M. Garner, Foued Salmen Espindola, Nilson Penha-Silva

**Affiliations:** 1 Institute of Biotechnology, Federal University of Uberlandia, Uberlandia, MG, Brazil; 2 Department of Physiology and Morphology, School of Medicine of ABC, Santo Andre, SP, Brazil; 3 Federal University of Acre, Rio Branco, AC, Brazil; 4 Post-Graduate Program in Physical Therapy, UNESP, Presidente Prudente, SP, Brazil; 5 Cardiorespiratory Research Group, Department of Biological and Medical Sciences, Faculty of Health and Life Sciences, Oxford Brookes University, Headington Campus, Oxford, United Kingdom; Nottingham Trent University, UNITED KINGDOM

## Abstract

Exhaustive exercise in a hot environment can impair performance. Higher epinephrine plasma levels occur during exercise in heat, indicating greater sympathetic activity. This study examined the influence of exercise in the heat on stress levels. Nine young healthy men performed a maximal progressive test on a cycle ergometer at two different environmental conditions: hot (40°C) and normal (22°C), both between 40% and 50% relative humidity. Venous blood and saliva samples were collected pre-test and post-test. Before exercise there were no significant changes in salivary biomarkers (salivary IgA: p = 0.12; α-amylase: p = 0.66; cortisol: p = 0.95; nitric oxide: p = 0.13; total proteins: p = 0.07) or blood lactate (p = 0.14) between the two thermal environments. Following exercise, there were significant increases in all variables (salivary IgA 22°C: p = 0.04, 40°C: p = 0.0002; α-amylase 22°C: p = 0.0002, 40°C: p = 0.0002; cortisol 22°C: p = 0.02, 40°C: p = 0.0002; nitric oxide 22°C: p = 0.0005, 40°C: p = 0.0003, total proteins 22°C: p<0.0001, 40°C: p<0.0001 and; blood lactate 22°C: p<0.0001, 40°C: p<0.0001) both at 22°C and 40°C. There was no significant adjustment regarding IgA levels between the two thermal environments (p = 0.74), however the levels of α-amylase (p = 0.02), cortisol (p<0.0001), nitric oxide (p = 0.02) and total proteins (p = 0.01) in saliva were higher in the hotter conditions. Blood lactate was lower under the hot environment (p = 0.01). In conclusion, enduring hot temperature intensified stressful responses elicited by exercise. This study advocates that hot temperature deteriorates exercise performance under exhaustive stress and effort conditions.

## Introduction

Physical activity induces physiological adjustment to support bodily changes during exercise. This adjustment varies with the duration [1], types and intensity of exercise [2], training level [3] and environmental conditions [4]. The analysis of salivary components such as total protein, α-amylase, immunoglobulin A (IgA), nitric oxide (NO) and cortisol may signify a non-invasive technique to determine the relationship of the intensity, duration, temperature, relative humidity and type of exercise with the changes that these situations could cause on the immune system and on the physical stress of the athlete [4–6].

Several studies have investigated the effects of exercise in different situations on the immunological system by salivary IgA and have reported decreased [7], increased [8] or unchanged [9] IgA levels post exercise. A scientific investigation demonstrated that a 100-km ultra-marathon induced negative immunological changes [10]. As a consequence, the authors recommended that exhaustive physical exercise would cause increased vulnerability to infections [10].

In this way, the stress response induced by exercise can be evaluated through the activity of salivary α-amylase [6, 11, 12], which is regulated by the adrenal sympathetic system, by means of the action of norepinephrine on the salivary glands. The physical and psychological strain generated by exercise stimulates the release of a glucocorticoid hormone cortisol by the adrenal gland [13,14], promoting mood deviations and decreased athletic performance [1,14]. This is because the increase in cortisol is bound to decreased action of serotonin in the brain, by lessening of mRNA coding for the synthesis of this neurotransmitter receptor [15]. The abovementioned studies support the analysis of levels of α-amylase and cortisol in saliva as reliable parameters to estimate the stress induced by exercise [1,6].

The stress response is connected with exercise intensity, which can be analyzed through lactate. Blood lactate levels are useful to determine the critical intensity of physical exercise tolerance or alternatively to assess the level of athletic training [16]. The increase in salivary levels of total protein during exercise is attributable to activation of the sympathetic nervous system [17,18] and thus expresses the level of exercise-induced stress.

Prolonged exercise commenced in a hot environment can impair the subject’s performance [19], as higher plasma concentrations of epinephrine during exercise elicited by heat induces higher sympathetic activity [20]. It has already been demonstrated that moderate-intensity exercise in a hot environment induced inflammatory processes [21] and that blood lactate responses to submaximal and maximal exercises are decreased under cool (10°C) or hot (35°C) conditions in soccer players [22]. Accordingly, it was recommended that athletes train in the morning during hot conditions, indicating the impact of hot temperature on immunological variables [23].

Declined salivary IgA accompanied by increased salivary α-amylase was reported in athletic runners during completion of an ultramarathon performed in hot conditions [24]. Recently, a study suggested that heat stress acts as a single stressor distinct from exercise [25].

In this manner, the research literature suggests that exercise in hot environments results in increased physiological stress [4,6,23–25]. Nonetheless, it was unclear whether there was an effect of hot temperature on salivary proteins, NO, IgA and α-amylase responses induced by exhaustive exercise. Here, this study aimed to compare stressful responses to exercise between a hot environment (40°C) compared to normal conditions (22°C) and between 40% and 50% relative humidity under both situations, by analysis of salivary biomarkers and blood lactate.

## Methods

This investigation was approved by the Ethics Committee in Research of the Federal University of de Minas Gerais (COEP 355/05/Brazil). This study was performed in accordance with the National Research Act of 1974 (P.L. 93–34). The research was directed in the Exercise Physiology Laboratory, located at Center of Excellence in Sports Science, School of Physical Education, Physiotherapy and Occupational Therapy, Federal University of Minas Gerais. The participants signed terms of informed consent. We examined nine healthy competent non-athletes and physically active men (24.2 ± 2.5 years old, 48.07 ± 4.63 mL_∙_kg^-1^_∙_min^-1^) enrolled in the Physical Education course at the Federal University of Minas Gerais. The profile of the sample population was based on regular physical activity and their physiological status (fat percentage, oxygen consumption-VO_2max-_, mass, height and body mass index-BMI). VO_2max_ was recorded during a maximal effort test with a bicycle ergometer three to five days prior to the experiment. Expired gases were analyzed to obtain the VO_2max_ recognized as the highest oxygen consumption attained during the protocol. Fat percentage was recorded via tetrapolar bioimpedance analysis. An inclusion criterion of VO_2 max_ between 40 and 60 mL∙kg^-1^∙min^-1^ was adopted.

### Procedures

Each subject accomplished two sessions of progressive maximal exercise on a bicycle ergometer, one in a hot environment (40°C) and another in a normal environment (22°C), both at between 40% and 50% relative humidity. The tests were completed at intervals of between three and five days to minimize any type of adaptation (training effects) during the test, and at the same time of day (from 15:00 to 16:00) to standardize influences of the circadian rhythm [23]. This period of interruption permits all biochemical variables analyzed to re-establish during the second protocol. Identical clothing was worn throughout all test conditions. The participants were instructed not to drink alcohol or beverages containing caffeine and, not to perform vigorous physical activity 24 hours prior to the test. Participants were not allowed to ingest any water during the trials.

Throughout the test period, volunteers were requested to ingest at least 500 ml of water two hours prior to the tests to ensure hydration status according to Armstrong [26]: specific gravity of less than or equal to 1.030. The urinary density was recorded before the start of the exercises to ensure that the volunteers were properly hydrated and afterwards, through a refractometer (JSCP—Uridens, São Paulo, SP, Brazil), previously calibrated with distilled water. The subjects were asked to maintain their usual diet and report their meals the night before and at breakfast the day of the test.

### Progressive exercise protocol

The tests commenced with a power equivalent to 60W and were increased by 15W every three minutes of exercise until fatigue. The rhythm was kept at 60 rpm. The peak power (WPeak22 and WPeak40) was computed according to the equation WPeak = W1 + (W2·t/180) [27], where W1 is the power corresponding the last complete stage, W2 is the power corresponding to the load increment of each stage and *t* is time in seconds for the duration of the incomplete stage. These ergo-spirometric variables were recorded simultaneously throughout the test. Saliva samples and, 25 μl of blood was taken from the earlobe prior to the start of exercise (pre-test) and at one (post-test 1) and five (post-test 5) minutes after exercise, for blood lactate inspection.

During the exercises, rectal temperature (Tr) was monitored continuously and logged every minute, and was considered as a gauge of the internal temperature (Ti). Measurements were completed with a disposable rectal probe (Yellow Springs, OH, 4491-E, USA), which was implanted approximately 11 cm beyond the anal sphincter and connected wirelessly (Yellow Springs, OH, USA).

The situations regarding test termination were: 1) request for cessation of exercise by the participant, 2) failure to maintain the pre-determined rhythm, 3) rectal temperature during exercise equal to or greater than 39.5°C, 4) occurrence of dizziness, confusion, pallor, cyanosis, nausea and/or signs of peripheral circulatory failure, and finally 5) acknowledgement of any complications with the equipment.

The participants were weighed undressed before and after completion of exercise, and total sweat rate was calculated as the difference in body mass, relativized by body surface area and divided by the time of exercise.

The urine specific gravity was measured before exercise, then, one minute and five minutes immediately post-test with the use of a refractometer (JSCP, Uridens, São Paulo, SP, Brazil) previously calibrated with distilled water. The participants were advised to maintain their usual diets and record the content of the meals taken ​​the night before and for breakfast on the day of test.

### Salivary analysis

Before saliva collection, the participants completed an oral antisepsis for the cleaning of cellular debris and other impurities. Salivation was stimulated by chewing one tablet of parafilm (Parafilm) with a mass of 0.5 grams. Chewing the tablet was coached in a recognized manner, without concern regarding speed, strength or frequency of chewing. The saliva collection was started immediately after the tests and the collection time was that attained in 1 minute. Saliva samples were placed in pre-cooled (4°C) mini-tubes and stored at -20°C for the analysis of α-amylase, total proteins, NO, IgA, and cortisol. All analyses were undertaken in duplicate. Analysis of the α-amylase activity was achieved by a kinetic method at 405 nm using the substrate 2-chloro-p-nitrophenyl-D-α-maltotrioside (CNP-G3) according to manufacturer's protocol (Amilasa 405, Wiener Lab, Argentina).

The total protein determination was decided using the biuret colorimetric method (UCFS DIASYS, Germany), with absorbance readings at 604 nm and 700 nm (Autoanalyser Architeet c8000, Abbot, IL, USA). The amount of salivary NO was measured by a colorimetric technique (Granger, Taintor, Boockvar, Hibbs, 1996) with an absorbance reading at 570 nm in a microplate reader (Titertek Multiskan Plus MK11). Datasets were analyzed using Microplate Manager application v4.0 (Bio-Rad Laboratories, USA). Analysis of total IgA was undertaken by an adaptation of the ELISA test (Mackinnon Hooper, 1994). Microtiter plates (Maxi-Sorp, Nunc, Wohlen) were sensitized with anti-human IgA (Sigma Chemical, Buchs) diluted in 0.06 M carbonate buffer pH 9.6 for 12 hours at 4°C. The plates were washed and blocked with orthophenylenediamine dihydrochloride (OPD). Saliva samples were diluted at a ratio 1:2 in 1% BSA-PBS buffer and incubated for 1 hour at room temperature. After washing, a biotinylated anti-IgA conjugate labeled with peroxidase was added to the solution. After exercise addition of peroxidase substrate (H_2_O_2_), diluted in the chromogen buffer (OPD), the solution was incubated at room temperature for 1 hour. For individual analysis of the plates, the results were stated as ELISA indexes (IE). The optical density (OD) values at 405 nm were reached in a microplate reader. After exercise obtaining the IgA concentrations, they were divided by the concentration of total proteins in the saliva to acquire the values of specific IgA. Measurement of cortisol was completed using a commercially available kit (Salimetrics, USA) and determination of optical density at 450 nm using an ELISA reader.

Salivary flow was collected before exercise, then, one minute and five minutes immediately post-test determined by dividing the volume by the saliva collection time.

### Blood tests

After local asepsis (absence of the microorganisms causing sepsis), samples of arterial blood (25 μl) were collected from the earlobe before exercise, then, one minute and five minutes immediately post-test, using glass capillaries containing heparin. Blood samples were transferred to mini tubes containing 50 μl of 1% sodium fluoride and stored at -20°C for analysis of lactate. The Lactate measurements were completed in duplicate by an electro-enzymatic method in an automated analyzer (YSI 2300 Sport L-lactate analyzer, USI Inc, Yellow Spring, Ohio, USA) [28].

### Statistical analysis

A normal distribution across the obtained results was assessed by the Shapiro-Wilk test. All datasets were expressed as mean ± standard deviation.

Comparisons of the variables between thermal environments (22°C vs. 40°C) and moments in time (pre-test vs. post-test 1 vs. post-test 5) were attained through the analysis of variance technique to model repeated measures on two factors scheme. Data from repeated measurements were checked for sphericity using the Mauchly test. Greenhouse-Geisser correction was applied when the sphericity was violated.

To consider the two phases (rest vs. recovery periods); the two-way ANOVA followed by the Bonferroni post-test for parametric distribution or, Friedman followed by the Dunn's post-test for non-parametric distribution were applied. Differences were considered significant when the probability of a Type I error was less than 5% (or, p<0.05).

To measure the extent of modification between phases for significant differences, the effect size was calculated using Cohen’s *d*. Large effect size was considered for values ≥ 0.9, medium for values between 0.9 and 0.5 and small for values between 0.5 and 0.25 [29].

GraphPad Prism 5 (GraphPad Software, San Diego, CA, USA) was used for statistical analysis.

## Results

### Anthropometric variables and exercise time duration

Table 1 presents data concerning age, mass, height, BMI, fat percentage and VO_2max_ of the volunteers.

**Table 1:** Mean values followed by their respective standard deviations of age, mass, height, BMI, fat percentage and VO_2max_.

Variables	
**Mass (Kg)**	74.99 ± 7.40
**Height (m)**	1.787 ± 0.4
**BMI (Kg/m**^**2**^**)**	23.66 ± 1.1
**Fat percentage (%)**	13.6 ± 5.8

BMI: body mass index; kg: kilogram; m: meters.

Time range of exercise was higher (p = 0.026, Cohen’s *d* = 1.15) at 22°C (33 ± 5.4 min) compared to 44°C (27 ± 4.97 min).

### Resting physiological values

**Fig 1:**
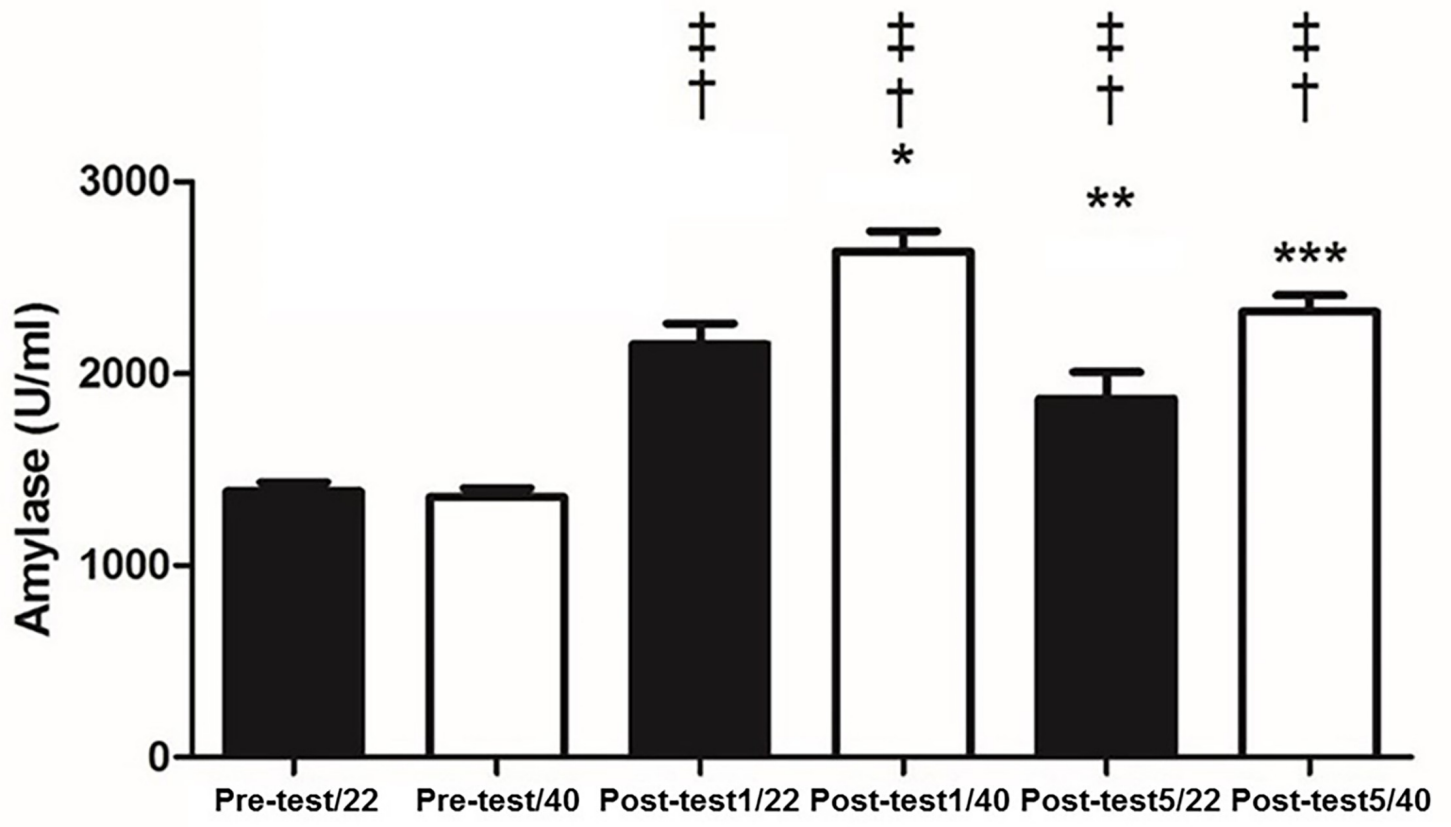
Activities of salivary α-amylase pre-test and at one (Post-test1) and five minutes (Post-test5) after physical exhaustion at temperatures of 22 and 40°C. † p < 0.05 different from Pre-test/22; ‡ p < 0.05 different from Pre-test/40; * p < 0.05 different from Post-test1/22; ** p < 0.05 different from Post-test1/40; *** p < 0.05 different from Post-test5/22.

**Fig 2:**
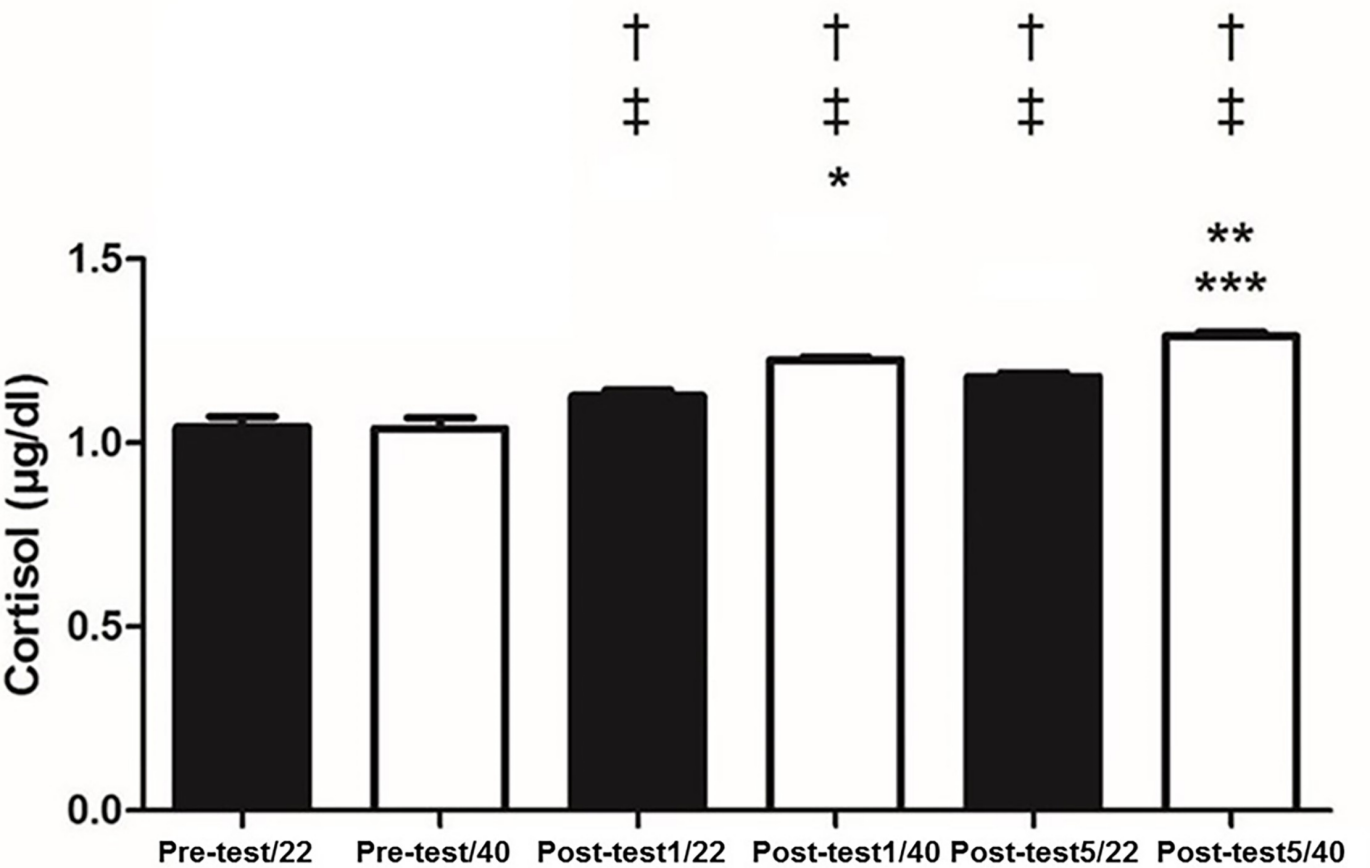
Salivary concentrations of cortisol pre-test and at one (Post-test1) and five minutes (Post-test5) after physical exhaustion at temperatures of 22 and 40°C. † p < 0.05 different from Pre-test/22; ‡ p < 0.05 different from Pre-test/40; * p < 0.05 different from Post-test1/22; ** p < 0.05 different from Post-test1/40; *** p < 0.05 different from Post-test5/22.

**Fig 3:**
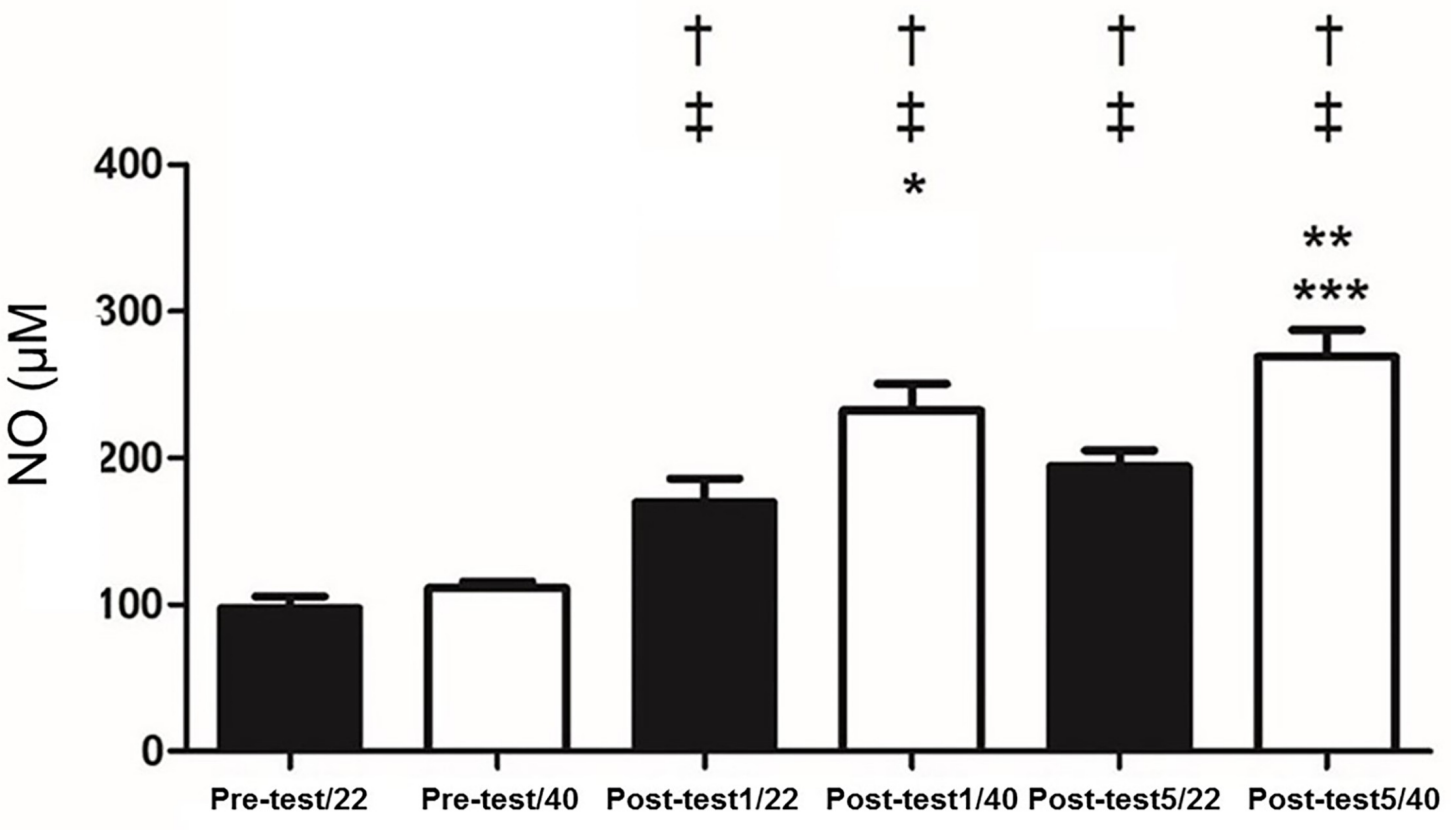
Salivary concentrations of nitric oxide pre-test and at one (Post-test1) and five minutes (Post-test5) after exhaustion at 22 and 40° C. † p < 0.05 different from Pre-test/22; ‡ p < 0.05 vs. Pre-test/40; * p < 0.05 different from Post-test1/22; ** p < 0.05 different from Post-test 1/40; *** p < 0.05 different from Post-test 5/22.

**Fig 4:**
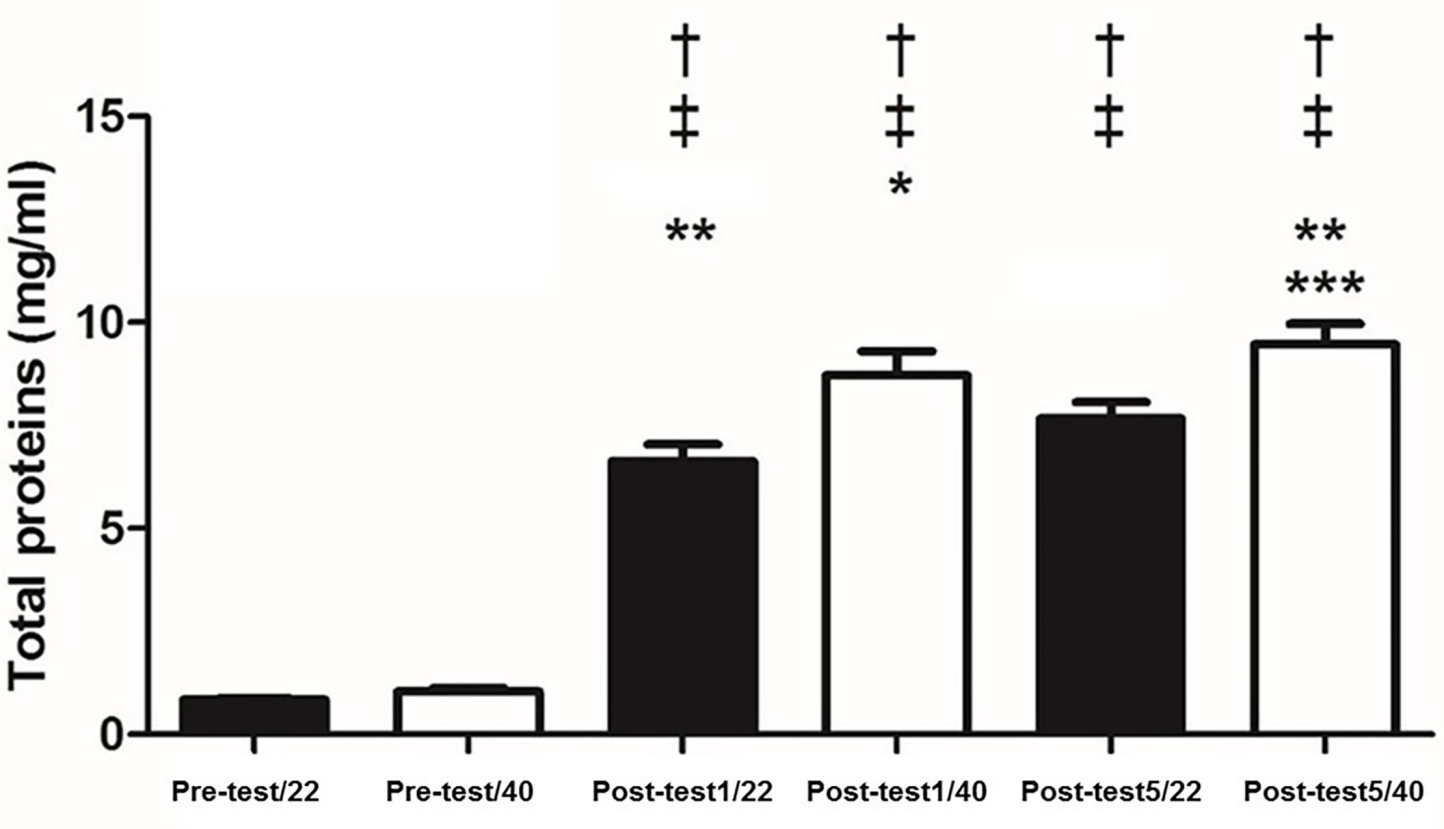
Concentrations of total salivary proteins pre-test and at one (Post-test1) and five minutes (Post-test5) after exhaustion at 22 and 40° C. † p < 0.05 different from Pre-test1/22; ‡ p < 0.05 different from Pre-test1/40; * p < 0.05 different from Post-test1/22; ** p < 0.05 different from Post-test1/40;*** p < 0.05 different from Post-test5/22.

**Fig 5:**
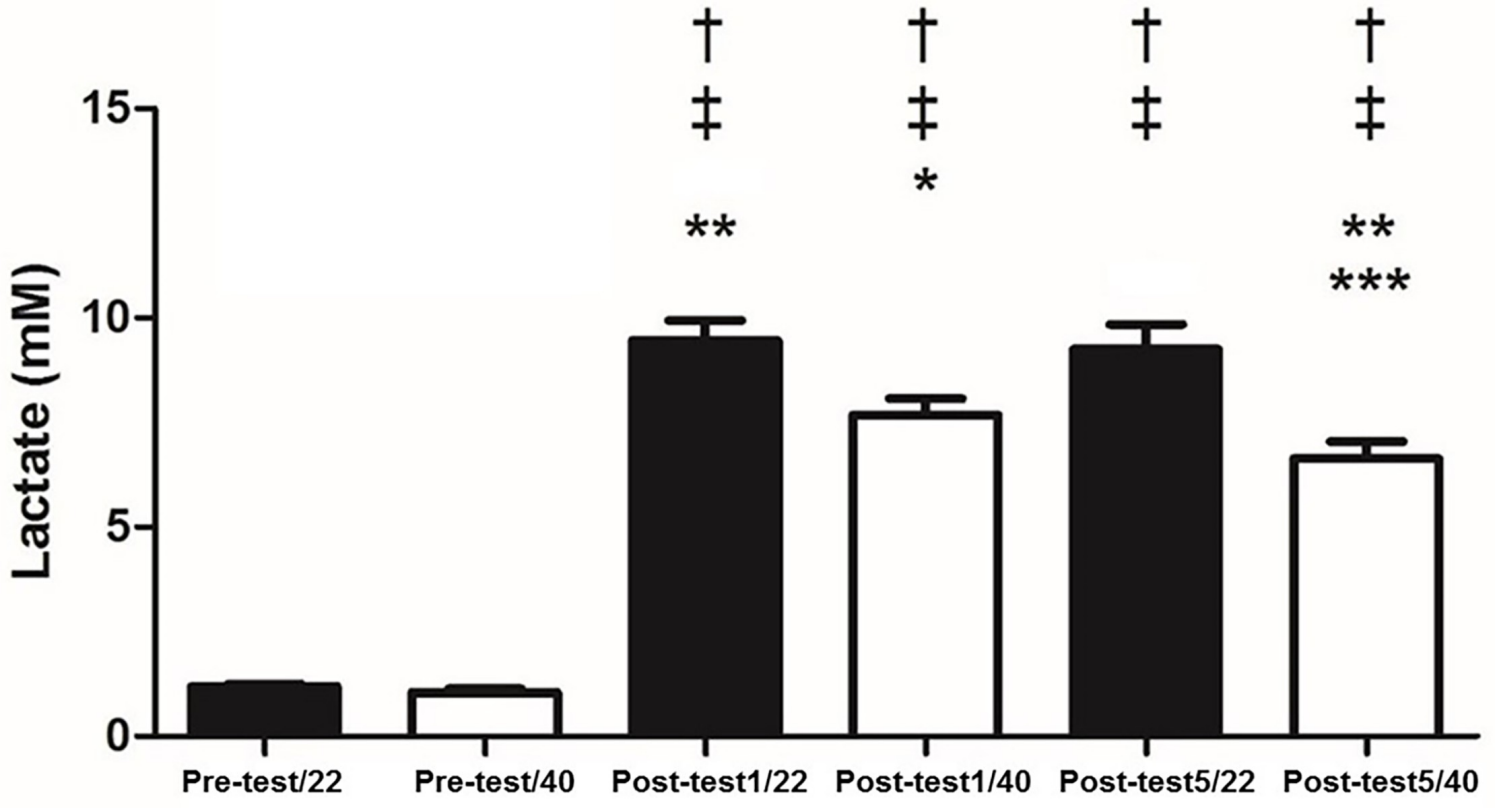
Blood lactate concentrations pre-test and at one (Post-test1) and five minutes (Post-test5) after exhaustion at 22 and 40° C. † p < 0.05 different from Pre-test/22; ‡ p < 0.05 different from Pre-test/40; * p < 0.05 different from Post-test1/22; ** p < 0.05 different from Post-test1/40; *** p < 0.05 different from Post-test5/22.

There were no changes between 22°C and 40°C, at rest for IgA (p = 0.15), salivary α-amylase (p = 0.4) (Fig 1), cortisol (p = 0.17) (Fig 2), NO (p = 0.07) (Fig 3), total proteins (p = 0.08) (Fig 4), or blood lactate (p = 0.16) (Fig 5).

Furthermore, we analyzed inter-individual difference of all variables. IgA ranged between 3.9 and 6.1 μg/dL, α-amylase between 1233 and 1583 U/mL, cortisol between 0.9 and 1.13 μg/dL, NO between 81 and 151.03 μM, total proteins between 0.708 and 0.987 mg/mL and finally lactate between 0.99 and 1.44 mM.

### Physiological responses induced by exercise

Post-test heart rate was increased at 40°C vs. post-test in 22°C (Cohen’s *d* = 13.03) and rest in 40°C (Cohen’s *d* = 10.9) (Table 2).

**Table 2:** Mean values followed by their respective standard deviations of heart rate (bpm) pre-test and post-test in 22°C and 40°C protocols.

Protocol	Pre-test	Post-test
**22°C**	69.778 ± 10.58*	184.78 ± 8.438*
**40°C**	80.333 ± 12.104	192.44 ± 8.079

*p<0.001 vs. post-test 40°C.

IgA concentrations increased with exercise at both temperatures. It was observed that α-amylase increased with exercise at both temperatures (Fig 1). Salivary cortisol increased post-test at both temperatures (Fig 2). NO levels increased post exercise (Fig 3). Total proteins concentration in saliva increased post exercise at both temperatures (Fig 4). Blood lactate levels increased post exercise (Fig 5) (Table 3).

**Table 3:** Cohen’s d regarding blood variables.

Variable	22°C—Pre-test vs. Post-test 1 min	22°C—Pre-test vs. Post-test 5 min	40°C—Pre-test vs. Post-test 1 min	40°C—Pre-test vs. Post-test 5 min
**IgA**	2.78*	0.18*	1.65*	0.8*
**α-amylase**	3.39*	2.09*	7.35*	6.99*
**Cortisol**	1.29*	2.25*	3.21*	4.26*
**NO**	2.27*	2.968	0.32*	0.41*
**Total proteins**	6.51*	8.63*	6.09*	7.7*
**Lactate**	8.15*	6.28*	8.21*	6.18*

**NO:** Nitric oxide; IgA: immunoglobulin A.

*p<0.05.

### Influence of temperature on physiological variables

IgA was unchanged between the 40°C and 22°C protocols (p>0.05). In contrast, it was observed that α-amylase was greater one minute (p<0.05; Cohen’s *d* = 7.32) and five minutes (p<0.05; Cohen’s *d* = 2.63) immediately post-test; salivary α-amylase was greater at 40°C compared to 22°C (Fig 1). Cortisol was significantly greater at 40°C compared to 22°C one-minute (p<0.05; Cohen’s *d* = 2.8) and five minutes (p<0.05; Cohen’s *d* = 3.95) post-test (Fig 2). NO levels were significantly higher at 40°C, one-minute (p<0.05; Cohen’s *d* = 0.13) and five minutes (p<0.05; Cohen’s *d* = 0.17) post-test compared to 22°C (Fig 3). Total protein was significantly greater at 40°C, one-minute (p<0.05; Cohen’s *d* = 1.36) and five minutes (p<0.05; Cohen’s *d* = 1.33) post-test compared to 22°C (Fig 4). Lactate was decreased at 40°C one-minute (p<0.05; Cohen’s *d* = 1.39) and five minutes (p<0.05; Cohen’s *d* = 1.68) post-test compared to 22°C (Fig 5).

## Discussion

This study aimed to control relevant variables so as to authenticate the effects of hot temperature on physiological stress responses to exhaustive exercise. Humidity, ambient temperature and body temperature were strictly controlled. As the main conclusion, it was demonstrated that hot temperature intensified stress responses to maximal exercise. However, no differences in IgA concentrations were observed between the environmental conditions. It was revealed that exercise duration was longer in the 22°C compared to 40°C protocol, we believe that it is possible that duration is relevant to the magnitude of the physiological response. However, the differences in physiological variables were evident despite reduced exercise time in the heat.

According to the current results, a significant increase of IgA in saliva post-test was observed. Yet, there was no significant impact of hot temperature on IgA responses. As the levels of IgA in saliva are reflective of the ability of immune system to protect [8], the results of this study suggested no decline in the immune capacity of saliva post-test performed at different levels of thermal stress. Nevertheless, generalization of the complete immune system based only on IgA should not be assumed.

IgA has been previously investigated in other studies that exposed increased levels of IgA after performing progressive exercise to exhaustion at different intensities and in the same thermal environment [8], and likewise after directing exercises at the same intensity to exhaustion in a hot environment (30.3 ± 0.1°C and 70% relative humidity) [4]. Until now, a decrease was conveyed in the levels of IgA in saliva in a moderate environment after performing a triathlon [30]. Taken together, the current findings suggest that hot temperatures do not intensify IgA reduction in response to exhaustive exercise.

It is of interest that such inconsistencies may be attributable to deviations in hydration status of athletes. Additionally, Blannin *et al* [8] reported that salivary IgA concentration and ratio to osmolality simultaneously increased during exhaustive exercise on an electrically braked cycle ergometer. As an important conclusion, the authors [4] indicated that exercise impacts the quantity but not the quality of saliva. Also, in this study, the state of hydration of the participants was suitably assessed before and after exercise by analyzing the urine specific gravity, which was always less than 1.030 [31].

Equally, decreased IgA and salivary flow has been previously observed during prolonged exercise in hot environments, and this was described as because of increased sympathetic activity [32,33]. These deliberations predict the existence of an inverse connection between the levels of cortisol and IgA, which was unnoticed in this study, since the elevation in the levels of IgA was lagged by increased levels of cortisol. These results agree with Laing *et al* [4], who found no inverse association between IgA and cortisol levels. But, the study by Laing *et al* [4] should not represent an appropriate basis of comparison with the results described in the current investigation, since the intensity of exercise in that study was smaller to that applied here. In this study, the participants performed exercise under increasing loads until exhaustion, while in the study of Laing *et al* [4] the exercise was accomplished under the same intensity for a fixed two hours. Despite the discrepancies in relation to the work of Laing *et al* [4], a significant increase in cortisol levels in the saliva in a hot environment coincides with other studies [1,34].

Whilst hot temperatures did not significantly influence IgA responses, it intensified salivary cortisol responses to exhaustive exercise, indicating increased stress responses. It should be consistent with the greater systemic stress induced by heat, which can then lead to decreased athletic performance [35]. The occurrence of a higher level of stress in a hot environment must have been the factor responsible for the more intense elevation of salivary α-amylase in a hot environment. This since α-amylase has been applied as a biomarker for the amount of stress produced by exercise [6,36], as its increase in saliva during exercise results from increased sympathetic activity of the β-adrenergic receptor [37,38].

Cortisol and α-amylase presented opposite responses compared with the anaerobic threshold marker (blood lactate concentration), since lactate was lower after exhaustive exercise in a hot environment, signifying increased muscle fatigue. This is in agreement with No *et al* [22], who evaluated male varsity soccer players submitted to bicycle exercise until exhaustion under three different conditions (10 ± 1°C, 22 ± 1°C and 35 ± 1°C). These researchers detected that blood lactate was reduced during maximal exercise at cool (10 ± 1°C) and hot (35 ± 1°C) conditions compared with warm environment (22 ± 1°C).

The key increase in blood concentration of lactate in a normal temperature environment (22°C) and total salivary proteins in the hot environment coincides with the results of studies performed in temperate [5,6] and hot environments [9]. As the total proteins increasing in saliva is also a consequence of activation of the sympathetic nervous system [18,19], this stimulation should have been more intense in the hot than the temperate environment [32].

Besides the stressful effect of hot environment in exhaustive exercise evidenced by higher levels of cortisol, α-amylase and total proteins, NO response to exercise was also increased at 40°C. Increased levels of NO with exercise concur with studies exhibiting an increase of NO after the performance of different types of physical activity at room temperature [38]. The increase of NO is related to blood vessel erosion, caused by the increase in systolic blood pressure [39], and, by an increase in activity of NO synthase (NOS) in proportion to exercise intensity and the enlarged vasodilatation because of the need for heat exchange with the surroundings [39]. The highest elevation in the levels of NO in a hot environment suggest a higher activity of NOS in this situation.

This current study suggests some points worth highlighting. Effect size calculation displayed the magnitude of the difference between protocols and moments, strengthening statistical significance of the current results. Body fat percentage and VO_2max_ were logged to standardize the study population. Higher exercise duration at 22°C removes the influence of time to exhaustion in the hot environment protocol.

A limitation of this study is the absence of data collection in a real environment. A real environment would influence some of these variables. As it was conducted under laboratory settings, this investigation rejected these potential external influences. This study did not measure autonomic function, which would provide additional information regarding physiological stress. Future studies are suggested to perform this specific kind of analysis. We did not measure hydration levels after exercise because the volunteers were unable to micturate.

Another limitation of this study is that the duration of exposure that was extended in the 22°C group compared to 40°C protocol; the period to exhaustion during maximal exercise was reduced in the hot environment. This needs highlighting because in reality triathletes, marathoners, and most endurance sportspersons exceed a period of 60 minutes when exercising.

The results of this investigation draw attention to subjects submitted to exhaustive exercise in hot environments. Considering that it increased the physiological stress, the sports medicine clinical team should be vigilant in hot temperature situations throughout competitions.

## Conclusion

Hot temperature intensified responses of cortisol, α-amylase, NO and total protein induced by exhaustive exercise, signifying more intense stressful responses elicited by the heat. Yet, there was no significant influence on IgA. These findings suggest that hot temperatures reduce exercise performance and increase the probability of disorders caused by maximal effort. It is therefore necessary to highlight the significance of this information to clinical sport coaches responsible for athletes during competition in hot environments.

## Supporting information

S1 File HR and exercise time. (XLSX)

S2 File Data. (XLSX)

S3 File Amylase. (XLSX)
