# Correction: The influence of a hot environment on physiological stress responses in exercise until exhaustion

**DOI:** 10.1371/journal.pone.0214627

**Published:** 2019-03-25

**Authors:** Romeu P. M. Silva, Cristiano L. M. Barros, Thiago T. Mendes, Emerson S. Garcia, Vitor E. Valenti, Luiz Carlos de Abreu, David M. Garner, Foued Salmen Espindola, Nilson Penha-Silva

The following information is missing from the Funding statement: This study received financial support from CNPq (308965/2015-9) and FAPESP (number 2018/26808-8 and 2015/19922-0) and Capes.
